# A Novel Approach of Determining the Risks for the Development of Hyperinsulinemia in the Children and Adolescent Population Using Radial Basis Function and Support Vector Machine Learning Algorithm

**DOI:** 10.3390/healthcare10050921

**Published:** 2022-05-17

**Authors:** Igor Lukić, Nevena Ranković, Nikola Savić, Dragica Ranković, Željko Popov, Ana Vujić, Nevena Folić

**Affiliations:** 1 Faculty of Medical Sciences, University of Kragujevac, 34000 Kragujevac, Serbia; igorukicvaljevo@gmail.com (I.L.); anavjc97@gmail.com (A.V.); nevena.folic@yahoo.com (N.F.); 2 Department of Computer Science, School of Computing, Union University, 11000 Belgrade, Serbia; 3 Faculty of Health and Business Studies, Singidunum University, 14000 Valjevo, Serbia; nikolasavicvzs@gmail.com; 4 Department of Mathematics and Statistics, Faculty of Applied Sciences in Nis, Union University “Nikola Tesla”, 18000 Nis, Serbia; nr17031994@gmail.com; 5 School Center with Dormitory “Dositej Obradović”, 24000 Subotica, Serbia; popovzeljko@gmail.com

**Keywords:** hyperinsulinemia, insulin, glucose, children and adolescents, risk factors, SVM

## Abstract

Hyperinsulinemia is a condition with extremely high levels of insulin in the blood. Various factors can lead to hyperinsulinemia in children and adolescents. Puberty is a period of significant change in children and adolescents. They do not have to have explicit symptoms for prediabetes, and certain health indicators may indicate a risk of developing this problem. The scientific study is designed as a cross-sectional study. In total, 674 children and adolescents of school age from 12 to 17 years old participated in the research. They received a recommendation from a pediatrician to do an OGTT (Oral Glucose Tolerance test) with insulinemia at a regular systematic examination. In addition to factor analysis, the study of the influence of individual factors was tested using RBF (Radial Basis Function) and SVM (Support Vector Machine) algorithm. The obtained results indicated statistically significant differences in the values of the monitored variables between the experimental and control groups. The obtained results showed that the number of adolescents at risk is increasing, and, in the presented research, it was 17.4%. Factor analysis and verification of the SVM algorithm changed the percentage of each risk factor. In addition, unlike previous research, three groups of children and adolescents at low, medium, and high risk were identified. The degree of risk can be of great diagnostic value for adopting corrective measures to prevent this problem and developing potential complications, primarily type 2 diabetes mellitus, cardiovascular disease, and other mass non-communicable diseases. The SVM algorithm is expected to determine the most accurate and reliable influence of risk factors. Using factor analysis and verification using the SVM algorithm, they significantly indicate an accurate, precise, and timely identification of children and adolescents at risk of hyperinsulinemia, which is of great importance for improving their health potential, and the health of society as a whole.

## 1. Introduction

### 1.1. Insulin and Glucose among Children and School Age Adolescents

In children and adolescents, various changes in the body occur during growth and development, due to the secretion of growth hormone. Since the main source of energy is glucose, there is an increased intake of foods rich in carbohydrates and sugars, most often unknowingly. This leads to increased secretion of the hormone insulin, which is not able to convert the increased amounts of glucose into the cells and thus remains in the blood. Elevated levels of insulin and glucose can be determined by laboratory tests. The causes of elevated levels of insulin and glucose do not have to be conditioned only by diet, but also by various other factors, such as poor physical activity, hereditary factors, and others. Initially, children and adolescents do not react to these elevated values, but over time, fatigue, thirst, hunger, weight gain, high blood pressure, high cholesterol, and more begin to appear. The role of insulin is to allow the cells in the body to take in glucose to use it as energy or to store it as body fat. If a disorder occurs, glucose builds up in the blood and this can lead to elevated blood sugar levels. If the body becomes resistant to insulin, it continues to produce more insulin than healthy children and adolescents produce, and then hyperinsulinemia occurs. Glucose levels gradually rise and lead to type 2 diabetes [1].

### 1.2. Reference Values of Insulin and Glucose among Children and School Age Adolescents

Normal blood glucose levels are 3.5 to 6.1 mmol/l, and insulin levels are 2.6–24.9 uIU/mL [2,3]. Initially, there are no pronounced symptoms of insulin resistance, which can sometimes not be manifested, and can occur only with an increase in blood sugar levels. The initial symptoms are reflected in the form of fatigue, hunger, loss of concentration, and nervousness. In the later stages, there is an increase in body weight, high blood pressure, and high cholesterol levels, until the stage when insulin resistance gradually turns into prediabetes, and then into type 2 diabetes [4,5]. If the blood glucose values are higher than 11.1 mmol/L after 120 min after the OGTT test, diabetes is diagnosed. If the blood glucose level after 120 min is from 7.7 mmol/L to 11.1 mmol/L, the diagnosis of glucose intolerance is made. If the blood glucose values fall below 7.7 mmol/L, this is a normal tolerance to glucose intake. It is normal that, after ingesting 75 g of glucose solution, blood glucose values increase at 30, 60, and 90 min, but return to reference values after 120 min. After the intake of glucose, the pancreas secretes a larger amount of insulin, which converts the glucose into energy, and the excess is stored in fat cells. After 2 h of glucose intake, insulin levels should be as close to baseline as possible, and certainly below 24.9 uIU/mL. In case the insulin values are elevated, depending on how high and whether the initial and final findings are higher, as well as whether the glucose values are normal or not, the diagnosis of insulin resistance is made [6,7], prediabetic conditions, or diabetes. Any change concerning the reference values, therefore, has an increased risk of developing more severe chronic non-communicable diseases, most often the development of type 2 diabetes, cardiovascular diseases, neuropathy, and others.

### 1.3. Consequences of Hyperinsulinemia

About 30% of such individuals develop type 2 DM (Diabetes Mellitus) within 10 years [2,8]. Different studies have shown that the most common risk factors are different. They largely depend on genetic predispositions, metabolic syndrome, the tendency to obesity, various hormonal disorders, poor physical activity, long-term consumption of certain drugs, alcohol, or other harmful substances [9,10]. Stress and poor nutrition are also cited as common factors, which greatly contribute to these changes in children and adolescents during their growth and development. To avoid or alleviate such a gradual disease, early diagnosis and preventive action are necessary [11]. Adolescence is the age of great physical and mental changes, which cause instability and oscillations in mood and behavior [3,12]. That is why it is extremely important to start monitoring and discovering possible risk factors at the earliest age, so that they could be eliminated in a timely manner. This is possible through the regular systematic examinations of children and adolescents in outpatient settings, calculation of BMI (Body Mass Index), measurement of blood pressure, and the monitoring of basic laboratory parameters, such as cholesterol, glucose, and other laboratory analyses. The aim of the study that will be presented in this paper is to identify the most influential risk factors for the development of hyperinsulinemia in children and adolescents at the earliest period of life. Once risk factors are identified, children and adolescents can prevent or mitigate further consequences. This is extremely important for each individual, as well as for general public health [3,13,14].

### 1.4. Goal of the Study

The aim of this paper is to find out why in certain regions in Serbia there is a higher percentage of children and adolescents with symptoms that indicate the appearance of hyperinsulinemia, which later results in type 2 diabetes and other diseases. The sample analyzed in this paper was not presented so far in other papers that we have published. It was noticed that in the municipalities of the Kolubara region there are children and adolescents who are: extremely obese; have a higher number of chronic diseases; and live in an environment that is economically more developed than other municipalities. We tried to use the appropriate methodology of statistical analysis and artificial intelligence tools to identify the factors that lead to this condition in children and adolescents.

Artificial neural networks were used to check the correctness of the methodology used in previous works, while in this paper the correctness, reliability, and accuracy of the model were checked using the SVM (Support Vector Machine) learning algorithm. The SVM algorithm is a reliable machine learning tool. Similar to factor analysis, it singles out more and less influential factors and separates them by a certain hyper level. The algorithm itself is used to check and compare the obtained results. It was shown that the children and adolescents in this sample are at higher risk by as much as 5% compared to the children and adolescents we have analyzed so far and then tested with various additional modern methods and techniques of artificial intelligence.

## 2. Previous Research

### 2.1. Previous Research Globally

The incidence of diabetes today in the world and in our country is a major public health problem. It is increasingly occurring in young and middle-aged people, with the most common being about 90% of type 2 diabetes. According to reports from the World Health Organization (WHO) and the International Diabetes Federation (IDF) from 2020. It is estimated that about 463 million people in the world suffer from diabetes, and it is predicted that by 2045 that number will increase to 700 million [15]. The highest incidence rate is registered in developed countries, but also in developing countries where Serbia belongs [16].

### 2.2. Previous Research in Serbia

According to the estimates of the Institute of Public Health “Dr Milan Jovanović Batut” in our country, about 750,000 people or 13.2% of the total population suffer from diabetes, of which about 95% have type 2 diabetes compared to type 1 diabetes [2,3,17]. Possible symptoms are increased sugar consumption, unusual weight gain, increased hunger and thirst, poor concentration, feelings of panic and anxiety, feeling tired, and more. Many types of research in the world and our country indicate that children and adolescents are at increased risk for proper growth and development. Various risk factors are also identified, that depend on many other conditions such as gender, race, geographical area, country of origin, and others [18,19]. Findings from several studies indicate that ectopic or visceral fat accumulation is associated with hyperinsulinemia, and that obesity is a significant risk factor for insulin resistance [20,21]. Research conducted with children and adolescents has shown that insulin resistance increases at the beginning of puberty, then reaches its maximum in the third phase, and then returns to prepubertal levels at the very end of puberty [22,23,24]. The consequence of these changes is the result of changes in growth hormones and insulin variations during the development of children and adolescents [25]. Previous research has shown that changes in insulin sensitivity occur as a result of fat accumulation. Recent research reveals that this phenomenon can be found in both lean and obese children and adolescents [26]. According to research by the International Diabetes Federation, increased obesity is an important risk factor for hyperinsulinemia [27]. Possible complications of hyperinsulinemia are: hypertriglyceridemia, hyperuricemia, atherosclerosis, obesity, hypertension, hypoglycemia, type 2 diabetes, normal growth disorder, sexual development, reproductive function, infertility, menstrual disorders, ovulatory dysfunction, coronary heart disease, and coronary heart disease [28,29]. In previous research by the same authors, a powerful tool of artificial intelligence, artificial neural networks (ANN), was used. Statistical processing of the data concerning the results obtained using ANN found a slightly lower level of risk in a significantly higher sample and differed by 0.4%. Using these two methods of factor analysis and the SVM algorithm, the authors showed accurate and precise results in both the degree of risk and in identifying risk factors for hyperinsulinemia [6].

### 2.3. Consequences of Hyperinsulinemia and Identified Risk Factors So Far

The consequences of hyperinsulinemia are numerous, starting with the risk of developing type 2 diabetes, cardiovascular diseases, and many others. The timely undertaking of all preventive measures at various degrees of risk is of exceptional importance, especially in the most sensitive specific period of each person’s life [30]. Children and adolescents at mild risk for this problem can eliminate or alleviate the symptoms by correcting their diet, increasing physical activity, changing their lifestyle, and improving their quality of life. Medium and higher risk requires the inclusion of additional pharmacological therapies to prevent further consequences [31,32]. Hyperinsulinemia is diagnosed using the OGTT and is based on insulin sensitivity index values. The research aims to determine the influence of various risk factors, such as obesity, poor nutrition, hereditary diseases, and others, on the occurrence of this problem [33,34].

## 3. Methodology

After the consent of the Ethics Committee of the Health Center in Valjevo, the research began in 2019 and was completed at the end of 2021. Subjects and their parents then agreed on the purpose and procedure of the research and provided written consent. In the first part of the research, during the regular systematic examination of children and adolescents, basic data were collected: gender, the environment they came from, assessment of general health, socio-economic living conditions, and more. Subjects then filled out a questionnaire that related to the inclusion in the diet of certain foods, beverages, diet, smoking habits, alcohol consumption or taking psychoactive substances, level of physical activity, and its impact on health [18,19]. The next part of the examination was performed by specialist doctors. Children and adolescents were measured for body weight, height, waist circumference, blood pressure, stress, family history, as well as basic hematological and biochemical analyses, and complete blood tests that included: glycemia, urea, creatinine, uric acid, alkaline phosphatase, direct bilirubin, total bilirubin, lipogram (K +, Na +, Mg ++), C-reactive protein, lipogram (cholesterol, HDL, LDL, triglycerides), ALT, and AST. Based on all the obtained analyses, the subjects were referred, according to the assessment of the pediatrician, to perform the OGTT test, if necessary [1,35].

Using the statistical program and the features of factor exploratory analysis, risk factors for the occurrence of this problem were determined. Principal component analysis (PCA) was performed, where the most dominant one that determines the degree of risk is singled out from a larger set of factors. Exploratory factor analysis (EFA) identified insulin as the leading factor in our study, which was to be expected. Confirmatory factor analysis (CFA) was used to test the hypotheses of a given model, whether there is a relationship between other selected factors and the variables that are analyzed in this way. Correlations between continuous variables were analyzed by Kruskal–Wallis, Chi-Square test, and pairwise test. In terms of probabilities: less than 0.05 were considered significant statistical differences. Mean values with standard deviation (SD) were calculated [36,37].

As a comparative model, the RBF function [38], within the IBM Statistical Package for the Social Sciences (SPSS, version 21.0), the Neural Network module, was used to monitor the influence of each risk factor on the overall risk of developing hyperinsulinemia. In addition, the SVM algorithm of machine learning was used, a robust and professional technique that minimizes error, and contributes to faster and more accurate monitoring of the impact of each risk factor. The SVM algorithm [39] divides the plane with the function f into two parts, so that the input risk factors lie above or below the function f. Significantly better results are expected using the RBF function, with the sigmoid activation function being chosen, and significantly more accurate and reliable results are also expected using the SVM algorithm [40,41,42].

## 4. Results

The sample in the presented research consisted of 674 children from the territory of six municipalities of the Kolubara district, which performed a regular systematic examination of school children. The study involved: 331 (49.1%) male respondents and 343 (50.9%) female respondents. Respondents came from: 244 urban areas (36.2%); 228 suburban areas (33.8%); and 202 rural areas (29.9%). The mean age of the subjects was 14.8 ± 1.7. After the OGTT test, the subjects were divided into two groups: the experimental group consisted of 117 (17.4%) subjects, who had elevated glucose and/or insulin levels and were at risk, and a control group of 124 (18.4%), which consisted of subjects with normal glucose values and/or insulin, or were not at risk. To calculate the most influential factor, confirmatory factor analysis was used, which determined that insulin has the most dominant effect, with a variance of 52.74%. The exploratory factor analysis singled out four factors that have the greatest impact on the risk of developing hyperinsulinemia. These are glucose with a variance of 23.28%, BMI with a variance of 15.80%, cholesterol with a variance of 13.71%, and hereditary factor with a variance of 11.17%.

Within the experimental group, the subjects were divided into three subgroups, based on the results of the OGTT test: a group of subjects at low risk, medium, and high risk.

Glucose at 0 min for group I was (4.5 ± 0.6 mmol/L), for group II (5.1±0.4 mmol/L), and for group III (6.4 ± 0.6 mmol/L). Insulin at 0 min for group I was (15.7 ± 3.5 μU/mL), for group II (23.7 ± 4.2 μU/mL), and for group III (30.4 ± 3.7 μU/mL). It can be concluded from the Kruskal–Wallis H test that there were no statistically significant differences between the groups at 0 min for either glucose or insulin (Table 1).

Glucose at 60 min for group I was (6.8 ± 0.5 mmol/L), for group II (7.7 ± 0.4 μU/mL), for group II (72.4 ± 20.5 μU/mL), and for group III (87.6 ± 12.2 μU/mL). Based on the Kruskal–Wallis X test, it can be concluded that there were no statistically significant differences in the values obtained for glucose in all three groups, but there were significant differences for insulin (Table 1).

**Figure 1:**
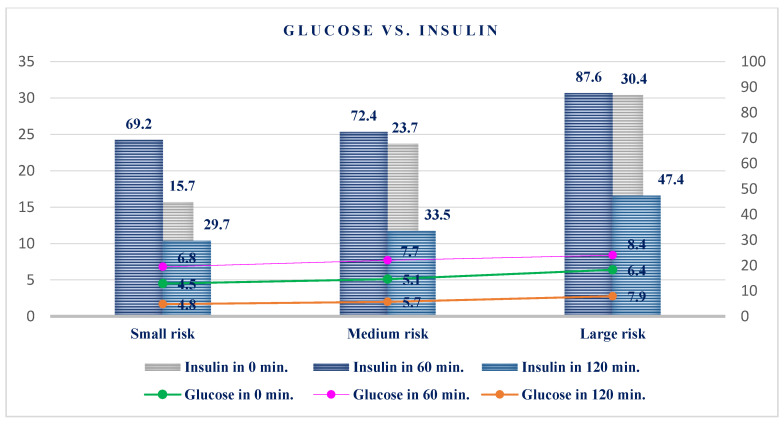
Glucose and insulin at 0, 60, and 120 min.

Glucose at 120 min for group I was (4.8 ± 0.4 mmol/L), for group II (5.7 ± 0.7 mmol/L), and for group III (7.9 ± 0.8 mmol/L). Insulin at 120 min had a value for group I (29.7 ± 19.8 μU/mL), for group II (33.5 ± 15.6 μU/mL), and for group III (47.4 ± 18.9 μU/mL). Based on the Kruskal–Wallis H test, in the third group there were significant differences for glucose, while for insulin there were significant differences in the first and second groups (Table 1, Figure 1).

HOMA-IR (Homeostasis Model Assessment of Insulin Resistance) index is calculated based on the value of blood glucose and insulin concentration, according to the formula:

(1) $$\mathrm{HOMA-IR}=\frac{Insulin\;in\;0\;min.\;\cdot \;Glucose\;in\;0\;min.}{22.5}$$

The assessment of the homeostatic model HOMA-IR is used to assess the function of the pancreatic beta cells and IR (Insulin Resistance) based on the values obtained by measuring the levels of basal (fasting) glucose and insulin. HOMA-IR in healthy individuals has a value of 1. Values from 1 to 1.6 represent peripheral insulin resistance, while values from 1.6 to 2 are defined as pre-hyperinsulinemia. HOMA-IR values greater than 2 are defined as hyperinsulinemia [33,34]. If the insulin values are elevated, and depending on the amount of glucose values, the diagnosis of insulin resistance, hyperinsulinemia, or even type 2 diabetes is made [35,36].

Out of a total of 117 (22.7%) respondents, 66 (9.8%) had HOMA-IR values of 1.0–1.6, of which 29 (4.3%) were male and 37 (5.5) %) of female subjects, and were considered to be at low risk for hyperinsulinemia. HOMA-IR values of 1.6–2.0 were found in 38 (5.6%) subjects, 17 (2.5%) men and 21 (3.1%) women, and they were at medium risk of pre-hyperinsulinemia. HOMA-IR values > 2 were found in 13 (1.9%) subjects, 5 (0.7%) male and 8 (1.2%) female subjects, where the presence of hyperinsulinemia could be diagnosed. The obtained percentage values refer to the sample N = 674, (Table 2).

The Kruskal–Wallis test shows significant statistical differences observed by the sex of the subjects within each group (KW (H) = 5.124, probability *p* = 0.023). There is a high percentage of children and adolescents with elevated BMI (KW (H) = 3.125, probability *p* = 0.068). but the value of the test shows that there are no statistically significant differences within each group. By analyzing the family anamnesis of the respondents, it can be concluded that there are no statistically significant differences between the respondents in all three of the observed groups (KW (H) = 1.305, probability *p* = 0.253), but that the percentage of respondents is high. When analyzing the poor nutrition, it can be concluded that there are significant differences in the subjects (KW (H) = 4.003, probability *p* = 0.048). Insufficient physical activity has a high percentage in many respondents, but there are no significant differences between them (KW (H) = 0.637, probability *p* = 0.412). When it comes to socio-economic conditions, there are no significant differences between the respondents of all three groups (KW (H) = 3.245, probability *p* = 0.071). Mental problems and stress are a smaller percentage of risk factors, but in all three groups, there are significant differences (KW (H) = 0.053, probability *p* = 0.813). Analyzing the consumption of psychoactive substances (PAS), there are no significant differences, but the largest percentage of respondents use alcohol and cigarettes (KW (H) = 3.517, probability *p* = 0.059). By measuring cholesterol, it was determined that there are significant differences (KW (H) = 4.237, probability *p* = 0.008). By measuring blood pressure in all three groups, significant differences were observed (KW (H) = 5.246, probability *p* = 0.026). Increased CRP is present in a high percentage of all respondents at risk and there are significant differences between them (KW (H) = 10.317, probability *p* = 0.001) (Table 3, Figure 1).

The research study consisted of a total of 117 (17.3%) respondents, divided into three groups: group I: 9.8% of them, who were at lower risk according to HOMA-IR, which is considered insulin resistance; group II: 5.6% of them, who were at medium risk, according to HOMA-IR, which represents pre-hyperinsulinemia, and group III: 1.9% of them, who were at high risk according to HOMA-IR, where hyperinsulinemia can be diagnosed. A total of 241 (35.8%) respondents performed the OGTT test. The remaining 124 (18.4%) subjects were the control group, and their glucose and insulin values were within normal limits (Table 3).

The results obtained by statistical analyses and factor exploratory and confirmatory analysis showed that: In the high-risk group, poor physical activity (92.3%) and poor nutrition (84.6%) have the greatest impact, as shown by the high cholesterol (76.9%). In the group of respondents with medium risk, the most influential factors are BMI (68.4%) and poor physical activity (71.1%), which are again reflected in high cholesterol (71.1%). In the group of respondents with the lowest risk, the most influential factors are BMI (51.1%) and hereditary factors (48.5%), which again result in elevated cholesterol (51.5%) The obtained percentage values refer to the number of respondents within group I, group II, and group III, (Table 4).

**Figure 2:**
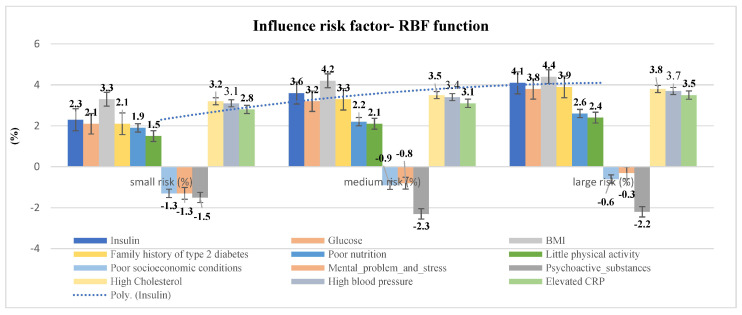
Risk factors for hyperinsulinemia—RBF function.

The results obtained using the RBF function module show the individual impact of each factor on the overall risk for the low, medium, and high risk groups for the development of hyperinsulinemia. In the high-risk group, BMI (4.4%) and hereditary factors (3.8%) have the greatest influence, as shown by the high cholesterol (3.8%). In the group of respondents with medium risk, the most influential factors are BMI (4.2%) and hereditary factors (3.3%), which are again reflected in high cholesterol (3.5%). In the group of respondents with the lowest risk the most influential factors are BMI (3.3%) and hereditary factors (2.1%), which again result in elevated cholesterol (3.2%) The obtained percentage values refer to the sample N = 674 (Table 5, Figure 2).

**Figure 3:**
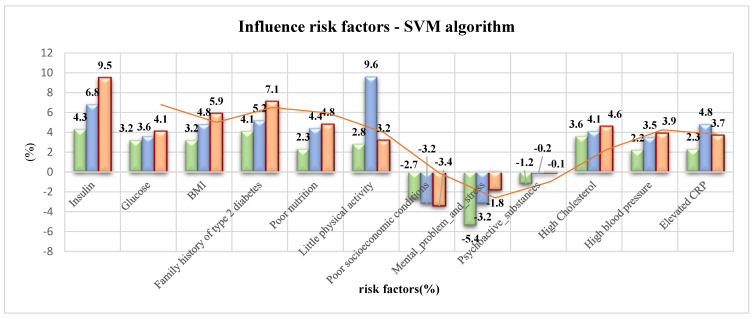
Risk factors—SVM algorithm. Green color: small risk for selected risk factor; Blue color: medium risk for selected risk factor; Orange color: huge risk for selected risk factor.

The results obtained using the SVM algorithms show the individual impact of each factor on the overall risk in the groups of low, medium, and high risk for hyperinsulinemia. In the high-risk group, BMI (5.9%) and hereditary factors (7.1%) have the greatest impact, as shown by high cholesterol (4.6%). In the group of respondents with medium risk, the most influential factors are BMI (4.8%) and hereditary factors (5.2%), which are again reflected in high cholesterol (4.1%). In the group of respondents with the lowest risk, the most influential factors are BMI (4.1%) and poor physical activity (2.8%), which again result in elevated cholesterol (3.6%) (Table 6, Figure 3).

## 5. Discussion

By analyzing the obtained results, it can be concluded that the children and adolescents are at different degrees of risk, divided into three groups. The first risk group has a low risk and has insulin resistance. Children and adolescents in the second group of the realized experiment are at medium risk and have pre-insulinemia. The third group of subjects is at the highest risk and can be diagnosed with hyperinsulinemia. It can be concluded that a total of 17.4% of the children and adolescents are at risk of developing hyperinsulinemia, which means that they can develop a chronic disease later in life, primarily type 2 diabetes, some cardiovascular diseases, and others. The conducted research showed that a higher percentage of the female respondents are at risk for developing hyperinsulinemia in all three of the observed groups compared to the male respondents. This is the case with the low-risk group, with 5.5% of female respondents compared to male respondents. In the medium-risk group, there are 3.1% of female respondents compared to 2.5% of male respondents. In the third group, there are 1.2% of high-risk female respondents and 0.7% of male respondents (Table 2). It can be concluded that the presented research on this sample showed the presence of risk factors at 5% higher compared to a similar previous research [6].

The Paired Samples Test (*t*-test), for the comparative analysis of glucose and insulin before and after the OGTT test, showed that there are statistically significant differences in the values of both glucose and insulin, which means that there is a risk. BMI shows the accuracy of the presented analyses, its influence is another significant risk factor. Hereditary factors represent a high percentage of the risks, alongside poor nutrition and the poor physical activity of the respondents, and they are present in all groups. Socio-economic conditions, psychological problems, stress, and the use of psychoactive substances are also present in a smaller percentage but do not have a large impact on the risk of disease. A high percentage of the respondents have high cholesterol, high blood pressure, and CRP. In the high-risk group, there are 76.9% of the respondents with high cholesterol, also 46.2% of the respondents with high blood pressure, and 69.2% of the respondents with high CRP (Table 3).

If similar studies are analyzed, it can be concluded that the percentage of children and adolescents at risk for hyperinsulinemia is different, largely depending on the level of development of the country in which the study is conducted. The presence of hyperinsulinemia and type 2 diabetes in the white population is still lower than in black and Asian adolescents [1,2]. The number of children and adolescents at low, medium, or high risk is increasingly coming from developing countries, including our country, which is why it is extremely important to monitor and analyze this problem, with mandatory preventive action [3,6].

It can be concluded that the use of reliable and proven methodologies is critical in determining the degree of risk where it can be of great diagnostic value for the adoption of corrective measures to prevent this problem and the development of potential complications. In addition, a reliable methodology such as factor exploratory and confirmatory analysis together with the verification of the obtained results; the SVM algorithm assessed the accurate and precise results obtained, and there were no differences in deviations.

It should be noted that the risk factor COVID-19 was not included in this study, because the beginning of the research started before the pandemic. Future research will aim to examine children and adolescents who have had COVID-19 infection and its impact on the development of hyperinsulinemia.

The entire research should examine the consequences of the growing presence of school-age children and adolescents with symptoms of hyperinsulinemia in Serbia. The first survey of 822 respondents published in 2021 found that 13.1% [6] of respondents are at risk of developing this problem. This study showed that this percentage is 17.4% in a similar population in other parts and municipalities of our country. Subsequent research should determine whether certain areas increase or decrease the risk of developing this problem.

## 6. Conclusions

The presented research showed that there are children and adolescents who are at a lower, medium, or high risk of developing hyperinsulinemia. Statistical analysis found that 17.3% of adolescents were at some degree of risk. It is clearly concluded that female adolescents are at higher risk than male children and adolescents, which can be explained by poor nutrition and much less physical activity. About 95% of the at risk children and adolescents have a higher BMI than normal, which indicates that obesity is mostly present in the analyzed children and adolescents, expressed during adolescence and significantly more in female than in male children and adolescents. Hereditary factors are present in a high percentage in all groups, which could be determined by using three different ways of examining the influence of risk factors. As a consequence of this condition, the respondents have a high percentage of elevated cholesterol. Insufficient factor at 120 min was isolated by factor exploratory analysis as the most influential indicator of the problem, and participates with 52.7% in the total risk. In addition, the confirmatory analysis identified three other significant risk factors, namely: BMI; hereditary factors; and poor physical activity. These claims were proven using two different ways of examining the influence of risk factors by RBF function and SVM algorithm. It is necessary for pediatricians and endocrinologists to make timely appropriate recommendations in the form of increased physical activity, changes in eating habits, and, depending on the individual, appropriate medication therapy.

## Figures and Tables

**Table 1:** Results of the OGTT test of the experimental group.

OGTT	Experimental Group					
N = 117 (17.4%)	I group 66 (9.8%)	II group 38 (5.6%)	III group 13 (1.9%)	$\chi^{2}$ and Kruskal–Wallis H		*p*
Glucose at 0 min (mmol/L) mean ± SD	4.5 ±0.6	5.1±0.4	6.4±0.6	KW(H)		
				I	0.003	0.936
				II	0.031	0.587
				III	0.724	0.396
Insulin at 0 min of OGTT (μU/mL) mean ± SD	15.7±3.5	23.7±4.2	30.4±3.7	KW(H)		
				I	0.006	0.953
				II	1.315	0.234
				III	0.135	0.718
Glucose at 60 min (mmol/L) mean ± SD	6.8±0.5	7.7±0.4	8.4±0.6	KW(H)		
				I	0.210	0.647
				II	0.179	0.672
				III	0.463	0.496
Insulin at 60 min of OGTT (μU/mL) mean ± SD	69.2±18.4	72.4±20.5	87.6±12.2	KW(H)		
				I	48.041	0.000 *
				II	27.063	0.000 *
				III	5.412	0.009 *
Glucose at 120 min (mmol/L) mean ± SD	4.8±0.4	5.7±0.7	7.9 ± 0.8	KW(H)		
				I	0.124	0.823
				II	3.249	0.075
				III	5.320	0.023 *
Insulin at 120 min of OGTT (μU/mL) mean ± SD	29.7 ± 19.8	33.5 ± 15.6	47.4 ± 18.9	KW(H)		
				I	4.735	0.028 *
				II	4.234	0.031 *
				III	0.273	0.575

Bold and *: ephasize the obtained results.

**Table 2:** HOMA-IR values.

HOMA-IR	1.0 < IR < 1.6	1.6 < IR < 2	IR > 2	Mean	Total
Male	29 (4.3%)	17 (2.5%)	5 (0.7%)	(2.50%)	(7.50%)
Female	37 (5.5%)	21 (3.1%)	8 (1.2%)	(3.30%)	(9.80%)
**Total**	**66 (9.8%)**	**38 (5.6%)**	**13 (1.9%)**	**(5.80%)**	**(17.30%)**

Bold: ephasize the obtained results.

**Table 3:** Risk factors for hyperinsulinemia.

Risk Factors	Small Risk	Medium Risk	Large Risk	Kruškal-Wallis H	*p*
Male Female	I group	II group	III group	5.124	0.023 *
	29 (43.9%)	17 (44.7%)	5 (38.5%)		
	37 (56.1%)	21 (55.3%)	8 (61.5%)		
BMI	34 (51.5%)	26 (68.4%)	9 (69.2%)	3.125	0.068
Family history of type 2 diabetes	32 (48.5%)	20 (52.6%)	7 (53.8%)	1.305	0.253
Poor nutrition	27 (40.9%)	24 (63.2%)	11 (84.6%)	4.003	0.048 *
Little physical activity	30 (45.5%)	27 (71.1%)	12 (92.3%)	0.637	0.412
Poor socioeconomic conditions	14 (21.2%)	13 (34.2%)	4 (30.8%)	3.245	0.071
Mental problems and stress	8 (12.1%)	9 (23.7%)	3 (23.1%)	0.053	0.813
Psychoactive substances	5 (7.5%)	7 (18.4%)	2 (15.3%)	3.517	0.059
High cholesterol	34 (51.5%)	27 (71.1%)	10 (76.9%)	4.237	0.008 *
High blood pressure	22 (33.3%)	19 (50.0%)	6 (46.2%)	5.246	0.026 *
Elevated CRP	23 (31.8%)	21 (55.3%)	9 (69.2%)	10.317	0.001 *

Bold and *: ephasize the obtained results.

**Table 4:** Influence risk factors.

BMI	51.5	68.4	69.2
Family history of type 2 diabetes	48.5	52.6	53.8
Poor nutrition	40.9	63.2	84.6
Little physical activity	45.5	71.1	92.3
Poor socioeconomic conditions	21.2	34.2	30.8
Mental problems and stress	12.1	23.7	23.1
Psychoactive substances	7.5	18.4	15.3
High Cholesterol	51.5	71.1	76.9
High blood pressure	33.3	50	46.2
Elevated CRP	31.8	55.3	69.2

**Table 5:** Influence risk factors—RBF function.

	Small Risk (%)	Medium Risk (%)	Large Risk (%)
Insulin	2.3	3.6	4.1
Glucose	2.1	3.2	3.8
BMI	3.3	4.2	4.4
Family history of type 2 diabetes	2.1	3.3	3.9
Poor nutrition	1.9	2.2	2.6
Little physical activity	1.5	2.1	2.4
Poor socioeconomic conditions	−1.3	−0.9	−0.6
Mental problems and stress	−1.3	−0.8	−0.3
Psychoactive substances	−1.5	−2.3	−2.2
High Cholesterol	3.2	3.5	3.8
High blood pressure	3.1	3.4	3.7
Elevated CRP	2.8	3.1	3.5

**Table 6:** Influence risk factors—SVM algorithm.

	Small Risk (%)	Medium Risk (%)	Large Risk (%)
Insulin	4.3	6.8	9.5
Glucose	3.2	3.6	4.1
BMI	3.2	4.8	5.9
Family history of type 2 diabetes	4.1	5.2	7.1
Poor nutrition	2.3	4.4	4.8
Little physical activity	2.8	9.6	3.2
Poor socioeconomic conditions	−2.7	−3.2	−3.4
Mental problems and stress	−5.4	−3.2	−1.8
Psychoactive substances	−1.2	−0.2	−0.1
High Cholesterol	3.6	4.1	4.6
High blood pressure	2.2	3.5	3.9
Elevated CRP	2.3	4.8	3.7

## Data Availability

The data used in this study are not publicly available and can be found upon request and approval from the Health Care Center in Valjevo.
